# Cascade Recovery of Bioactive Compounds from Spent Brewer’s Yeast Using Pulsed Electric Field Treatment and Subsequent Enzymatic Hydrolysis

**DOI:** 10.3390/microorganisms14081737

**Published:** 2026-08-07

**Authors:** Ralitsa Veleva, Valentina Ganeva

**Affiliations:** 1 Department of Cell Biology and Developmental Biology, Faculty of Biology, Sofia University “St. Kliment Ohridski”, 1164 Sofia, Bulgaria; ralitsa_veleva@biofac.uni-sofia.bg; 2 Department of Biophysics and Radiobiology, Faculty of Biology, Sofia University “St. Kliment Ohridski”, 1164 Sofia, Bulgaria

**Keywords:** irreversible electropermeabilization, biomass valorization, Alcalase, enzymatic hydrolysis, peptides, antioxidant activity, cell viability

## Abstract

Spent brewer’s yeast (SBY) is a protein-rich by-product of the brewing industry and a valuable source of intracellular nutrients and bioactive compounds. In the present study, a cascade extraction process was developed to obtain bioactive compounds from this biomass. Pulsed electric field (PEF) treatment in continuous-flow mode was applied to achieve irreversible plasma membrane permeabilization of the cells. Following PEF treatment, the primary water fraction containing released intracellular compounds was recovered, and the residual biomass was incubated with 0.2% (*v*/*v*) Alcalase for 6 h at 40 °C. The enzymatic treatment generated hydrolysates characterized by high levels of protein and free amino nitrogen and strong antioxidant activity. Ultrafiltration analysis revealed that the <3 kDa fraction retained approximately 78% of the recovered protein and 90% of the overall antioxidant capacity. The biocompatibility of the water extracts was evaluated using HepG2, HaCaT, and A375 cell lines. They were well tolerated and enhanced cell viability in HaCaT and A375 cells after 48 h exposure. These findings demonstrate the potential of the proposed cascade extraction process for more efficient utilization of SBY biomass through the generation of value-added products for nutraceutical, cosmeceutical, and pharmaceutical applications.

## 1. Introduction

Spent brewer’s yeast (SBY) is a major by-product generated by the brewing industry, accounting for approximately 15% of the total brewery waste. It is characterized by high protein content, typically ranging from 45 to 55% of the dry cell weight [1,2], and is also rich in low-molecular-weight bioactive compounds, including B-complex vitamins, minerals, free amino acids, glutathione, and other antioxidant constituents. The cell wall contains significant amounts of polysaccharides, mainly β-glucans and mannans, which are associated with various health-promoting properties. This complex composition makes SBY a promising raw material for the production of ingredients intended for nutraceutical, cosmeceutical, and pharmaceutical applications [3,4]. Nevertheless, despite its considerable biotechnological potential, SBY is still used predominantly as a low-value protein source in animal feed [5].

Consequently, increasing attention has been directed toward the development of sustainable strategies for its valorization through the recovery of high-value functional ingredients [1,2,3,4]. Among the various valuable constituents of SBY, proteins have attracted considerable interest due to their high abundance and potential as precursors of biologically active peptides [5,6,7,8]. The production of peptide-enriched yeast extracts is especially promising, as numerous studies have demonstrated their antioxidant, antihypertensive, immunomodulatory, and other bioactive properties [6,9,10,11,12].

The most common method for obtaining such extracts is enzymatic hydrolysis. Unlike traditional autolysis, it relies on selected enzymes, allowing better control over the process and the composition of the resulting products while ensuring high peptide yields [8,12]. Hydrolysis can be performed using a single exogenous endoprotease or combinations of endoproteases and exoproteases, enabling the efficient conversion of proteins into soluble peptides under relatively mild conditions [6,10,13,14]. Alcalase, Flavourzyme, Neutrase, Papain, and Protamex are among the most frequently used commercial proteases [5,13].

The main challenge associated with the enzymatic hydrolysis of yeast biomass is the cell wall, which restricts the access of exogenous proteases to the cell interior where the majority of cellular proteins is located. The yeast cell wall has a layered structure, and its permeability to macromolecules is largely determined by the outer layer, which consists of highly glycosylated, tightly packed mannoproteins [15,16]. The structural properties of the cell wall, including its thickness and permeability to macromolecules, are strongly influenced by both cultivation conditions (such as medium composition and temperature) and cell age [17]. SBY, which usually undergoes up to six repitching cycles, is characterized by increased cell wall rigidity and reduced permeability, limiting the access of exogenous proteases to intracellular protein substrates and reducing the efficiency of enzymatic hydrolysis [14]. Consequently, efficient enzymatic hydrolysis often requires additional treatments that either disrupt the cell wall or modify its structure to facilitate enzyme access to intracellular proteins.

Among the approaches used to improve enzymatic hydrolysis are mechanical disruption, autolysis, and pretreatment with cell wall-degrading enzymes [7,14,18,19]. Mechanical disruption releases intracellular proteins and makes them directly accessible to exogenous proteases; on the other hand, it causes extensive cell fragmentation, which complicates downstream separation and fractionation processes, and is associated with high energy consumption [20,21]. During autolysis, endogenous enzymes induce structural modifications of the cell envelope, facilitating both the release of intracellular proteins and the access of exogenous proteases to the cell interior. However, autolysis usually requires prolonged incubation, typically performed at elevated temperatures, and its efficiency depends strongly on the physiological state of the biomass and the process conditions. An alternative approach is the use of cell wall-degrading enzymes, but this increases process complexity and cost, and its effectiveness depends on the structural properties of the biomass.

Accordingly, a treatment capable of simultaneously eliminating the barrier function of the plasma membrane and increasing cell wall permeability would be highly attractive, as it could facilitate both the recovery of part of the water-soluble bioactive intracellular compounds and the subsequent enzymatic hydrolysis of the residual biomass, thereby enabling a more comprehensive valorization of SBY.

Pulsed electric field (PEF) treatment is a non-thermal, energy-efficient, and scalable technology that has attracted considerable interest in the recovery of bioactive compounds from microorganisms and plant tissues [22,23]. Its primary effect is associated with the generation of an additional transmembrane potential which, upon reaching a critical threshold specific to the cell type, results in a loss of plasma membrane barrier properties, a phenomenon commonly referred to as electroporation or electropermeabilization [24,25,26]. Depending on the electrical treatment conditions and the physiological characteristics of the cells, membrane permeabilization may become irreversible. Under such conditions, extensive leakage of ions and water-soluble intracellular compounds occurs, resulting in cell death [27,28].

Since irreversible electropermeabilization does not normally cause cell disruption in microorganisms and plant cells, the release of intracellular compounds is selective. Ions and small water-soluble molecules diffuse out rapidly, whereas the efflux of proteins and other macromolecules remains constrained by the cell wall and proceeds considerably more slowly [29,30,31,32,33]. Therefore, the efficiency of macromolecule recovery depends strongly on cell wall composition and structure. This limitation is particularly relevant for microorganisms such as microalgae and yeasts, which are increasingly recognized as valuable sources of proteins and bioactive peptides for food, feed, and biotechnology applications. In this regard, PEF treatment represents an attractive alternative to conventional mechanical disruption techniques because it can be readily applied to large biomass volumes in continuous-flow systems while generally requiring lower energy input than mechanical cell disruption.

Various strategies have been explored to improve the efficiency and rate of protein recovery. One of these involves combining PEF treatment with externally supplied lytic enzymes to increase cell wall porosity, thereby facilitating protein release. PEF treatment itself may induce structural changes in the cell wall of different microorganisms [34,35,36,37,38]. Such structural changes make even stationary-phase baker’s yeast and SBY cells more susceptible to lytic enzymes [33,34,39].

Another strategy is based on the application of PEF treatment to increase the susceptibility of microbial cells to exogenous proteases. Electrical treatment has been shown to enhance the enzymatic hydrolysis of fresh biomass from the microalga *Scenedesmus almeriensis* by enabling the entry of Alcalase and Flavourzyme into the cells [40]. PEF-based cascade biorefinery approaches have been proposed for the sequential recovery of multiple biomass fractions [41].

More recently, PEF treatment leading to irreversible electropermeabilization was shown to significantly enhance the enzymatic hydrolysis of baker’s yeast [42] and SBY [39]. Subsequent incubation of these permeabilized cells with Alcalase yielded extracts containing approximately half of the intracellular protein content, predominantly in the form of low-molecular-weight peptides and exhibiting strong antioxidant activity, without the need for cell wall-degrading enzymes. Although PEF treatment increases cell wall porosity, the release of intracellular proteins from permeabilized cells during incubation in water remains limited and relatively slow [30,39]. Consequently, many low-molecular-weight bioactive compounds can be extracted rapidly, whereas most proteins remain associated with the residual biomass. This creates favorable conditions for a cascade valorization strategy. Following PEF treatment, the water-soluble fraction can be separated from the biomass, while the retained protein-rich fraction can subsequently be converted into bioactive peptides through controlled enzymatic hydrolysis. Such an approach enables the generation of multiple value-added products from the same starting stream while maintaining a simple and rapid process scheme.

Therefore, the present study aimed to evaluate the feasibility of a cascade extraction strategy for SBY and to determine whether recovery of the water-soluble intracellular fraction affects the efficiency of subsequent Alcalase hydrolysis. In parallel, the effect of increasing the substrate-to-enzyme ratio was assessed to explore the possibility of reducing enzyme consumption and improving process economics.

## 2. Materials and Methods

### 2.1. Yeast Biomass

The SBY used in this study was kindly provided by Kamenitza AD (Haskovo, Bulgaria). Experiments were performed using *Saccharomyces pastorianus* biomass collected after the fourth and sixth repitching cycles. The biomass was harvested by centrifugation (1000× *g*, 10 min; ROTINA 380, Andreas Hettich GmbH & Co. KG, Tuttlingen, Germany), and the residual fermentation medium was removed by discarding the supernatant. The pellet was washed once with distilled water, resuspended in distilled water, and kept at room temperature for 1 h. Following a second centrifugation step (1000× *g*, 10 min), the biomass was resuspended in distilled water to obtain a final concentration of 60.50 ± 1.63 g dry cell weight per liter (g DCW/L). The conductivity of the suspension was then adjusted to 0.30 ± 0.02 mS/cm using a 0.25 M sodium phosphate buffer at pH 7.

### 2.2. PEF Treatment

Continuous-flow PEF treatment was carried out using a Hydropuls Mini generator (GBS-Elektronik, Radeberg, Germany) delivering monopolar rectangular pulses (2300 V, 10 A), as previously described [39]. Pulse duration and frequency were controlled by an arbitrary waveform generator (RIGOL DG1012, RIGOL Technologies Co., Ltd., Suzhou, China). The pulsing chamber with a working volume of 0.825 mL was equipped with two parallel stainless-steel electrodes separated by 0.4 cm. PEF treatment was conducted at flow rate of 140 mL/min, controlled by a peristaltic pump (Ismatec, Glattbrugg, Switzerland). During passage through the chamber, the cells were exposed to 10 pulses of 0.5 ms duration at a pulse frequency of 28.3 Hz and an electric field strength of 3.1–3.25 kV/cm. The total treatment time, calculated as the product of the number of pulses (n) and pulse duration (τ) according to the equation t = n × τ, was 5 ms. The residence time of the cells in the chamber was approximately 0.35 s. The outlet temperature was measured using a K-type thermocouple connected to a digital thermometer, and the sensor was positioned at the chamber outlet. The specific treatment energy per liter of cell suspension was calculated as previously described [33]. All electrical parameters were monitored online using an oscilloscope (Instek GDS 2064, Good Will Instrument Co., Ltd., New Taipei City, Taiwan).

### 2.3. Determination of Dead Cells and Irreversible Electropermeabilization

Dead cells before PEF treatment and cells with irreversibly permeabilized plasma membranes after treatment were quantified by propidium iodide (PI) staining. To assess cell viability before PEF treatment, 5 µL of a 0.5 mM PI solution prepared in distilled water was added to 50 µL of cell suspension. Following a 5 min incubation at room temperature, the samples were washed once with distilled water and examined under an epifluorescence microscope (L3201 LED, Microscopesmall, Shenzhen, China). Fluorescent cells were counted and expressed as a percentage of the total number of cells. The same procedure was used to assess irreversible membrane permeabilization after PEF treatment, except that PI staining was performed approximately 1 h later. The extent of irreversible permeabilization was expressed as the percentage of fluorescent cells relative to the total cell count.

### 2.4. Post-PEF Processing of Cell Suspension

Following PEF treatment, both treated and untreated (control) cell suspensions were processed in parallel under identical conditions for all subsequent analyses.

#### 2.4.1. Release of Water-Soluble Compounds

For analysis of the released water-soluble intracellular compounds, the cell suspensions were incubated at room temperature for 4 h without additional dilution. After incubation, the suspensions were centrifuged at 3000× *g* for 10 min. The resulting supernatants were analyzed immediately or stored at −20 °C until required. The remaining cell pellets were either frozen for subsequent use or stored overnight at 4 °C before enzymatic hydrolysis.

#### 2.4.2. Enzymatic Hydrolysis

Enzymatic hydrolysis was performed using Alcalase^®^ 2.4 L FG, kindly provided by Novozymes (Bagsværd, Denmark). The cell pellets obtained after centrifugation were resuspended in 50 mM K_2_HPO_4_ to restore the original suspension volume, corresponding to a biomass concentration of 60.50 ± 1.63 mg DCW/mL, and the flasks were equilibrated for 15 min at 40 °C. Alcalase was then added to obtain a final concentration of 0.2% (*v*/*v*). The suspensions were incubated at 40 °C with shaking at 100 rpm in a Biosan ES-20/40 orbital shaker for 6 h and subsequently centrifuged at 3000× *g* for 15 min to remove the residual cell biomass. The obtained hydrolysates were divided into two portions. One portion was subjected to thermal inactivation at 90 °C for 15 min and frozen at −20 °C until analysis. The second portion was frozen immediately at −20 °C without thermal treatment and was used for the analysis of non-heated hydrolysates. The heat-treated hydrolysates were further fractionated by sequential ultrafiltration using Vivaspin^®^ 20 centrifugal filter units with molecular weight cut-offs (MWCO) of 10 and 3 kDa (Sartorius, Göttingen, Germany). The hydrolysates were first centrifuged through the 10 kDa membrane at 3000 rpm for 30 min. The resulting permeate was subsequently subjected to ultrafiltration through a 3 kDa membrane under the same conditions.

### 2.5. Preparation of Cell Lysate

Cell lysates were prepared by mechanical disruption with glass beads (0.42–0.6 mm diameter; Sigma-Aldrich Chemie GmbH, Schnelldorf, Germany). Cell suspensions (2 mL), previously diluted two-fold with distilled water or 100 mM K_2_HPO_4_, were transferred to plastic screw-cap tubes and mixed with glass beads at a suspension-to-beads volume ratio of 2:1. Cell disruption was achieved by vortexing for nine 1 min cycles separated by 15 s cooling intervals, during which the tubes were kept on ice. Following this treatment, the lysates were centrifuged at 10,750× *g* for 5 min. The supernatants were collected and stored at 4 °C until analysis.

### 2.6. Light Microscopy

To assess the integrity and morphology of yeast cells following PEF treatment and subsequent enzymatic hydrolysis, untreated yeast cells (control) and PEF-treated cells recovered after enzymatic hydrolysis were resuspended in 50 mM K_2_HPO_4_. The samples were examined using a light microscope (L3201 LED, Microscopesmall, Shenzhen, China) equipped with a 100×/1.25 oil-immersion objective. Representative images were acquired using the integrated digital camera and LissView software (Version 6.1.4.1, Guangzhou Liss Optical Instrument Co., Ltd., Guangzhou, China).

### 2.7. Analytical Methods

Protein concentration was determined by the method of Lowry et al. [43] using bovine serum albumin as the calibration standard.

Free α-amino nitrogen (FAN) content was quantified by a colorimetric ninhydrin assay according to Lie [44]. The ninhydrin reagent consisted of 0.2% (*w*/*v*) ninhydrin dissolved in a mixture of DMSO and 0.25 M sodium acetate buffer (pH 5.5) at a ratio of 40:60 (*v*/*v*). Equal volumes (200 µL) of sample and ninhydrin reagent were combined in microcentrifuge tubes and heated at 90 °C for 15 min in a dry block heater. After cooling to room temperature, 1 mL of ethanol–water (1:1, *v*/*v*; prepared from 97% ethanol) was added, the samples were vortex-mixed, and absorbance was measured at 570 nm. The FAN concentration was calculated from a glycine calibration curve (10–100 mg/L) and expressed as mg glycine equivalents per gram of dry cell weight (mg GE/g DCW).

Antioxidant capacity was evaluated by the Trolox equivalent antioxidant capacity (TEAC) assay based on the ABTS radical cation according to Pellegrini et al. [45], with minor modifications. The ABTS radical cation was generated from a 7 mM ABTS solution containing 2.45 mM potassium persulfate. Before use, the solution was diluted with 50 mM potassium phosphate buffer (pH 7.0) to an absorbance of 0.70 ± 0.02 at 734 nm. Aliquots (5 µL) of undiluted samples or samples diluted two- to eight-fold were mixed with 995 µL of the working solution. The decrease in absorbance at 734 nm was measured after 15 min. Quantification was performed using a Trolox calibration curve in the range of 0.1–15 µM, and antioxidant activity was expressed as mg Trolox equivalents per gram of dry cell weight (mg TE/g DCW).

### 2.8. Effect of Water Extracts on the Viability of HaCaT Keratinocytes, A375 Melanoma Cells, and HepG2 Liver Cancer Cells

Cells were routinely maintained in 90 mm Petri dishes and cultured under standard conditions (37 °C, 5% CO_2_) in DMEM supplemented with 10% FBS and 1% (*v*/*v*) antibiotic–antimycotic solution containing 100 U/mL penicillin, 100 μg/mL streptomycin, and 0.25 μg/mL amphotericin B. For the cell viability experiments, the water extract obtained from PEF-treated cells was freeze-dried for 48 h and reconstituted in DMEM supplemented with 25 mM HEPES (pH 7.4). The resulting stock solution was sterilized by filtration through a 0.22 µm Durapore membrane filter (Millipore Co., Bedford, MA, USA). Cell viability was determined using the crystal violet assay [46]. After trypsinization, cells were seeded into 96-well plates at a density of 2 × 10^5^ cells/mL and allowed to recover for 24 h under standard culture conditions. The culture medium was then replaced with fresh medium containing the freeze-dried water extract at final dry matter concentrations of 0.35–5.60 mg/mL. Following 24 or 48 h of incubation, the cells were washed with PBS and fixed with 4% buffered formaldehyde for 20 min. The plates were rinsed with distilled water, stained with 1% crystal violet solution for 20 min, washed thoroughly to remove excess dye, and air-dried. The bound crystal violet was dissolved in 10% acetic acid, and absorbance was measured at 570 nm using an Epoch microplate spectrophotometer (BioTek Instruments, Winooski, VT, USA) operated with Gen5 software (Gen5^TM^ Data Analysis software, version 1.11.5). Cell viability was expressed as a percentage of the absorbance of untreated control cells.

### 2.9. Determination of Biomass and Dry Matter Content of the Extracts

Dry cell weight was determined by microwave drying as described by Rice et al. [47]. Aliquots of 1 mL of cell suspension were filtered through membrane filters (pore size 0.45 µm), and the filters containing the retained biomass were dried in a microwave oven to constant weight. Dry cell weight was calculated as the difference between the filter weight before and after filtration and drying.

The dry matter content of water extracts and hydrolysates was determined by drying the samples in pre-weighed glass vessels at 100 °C to constant weight. The amount of released dry matter was calculated as the difference between the vessel weights before and after drying. For hydrolysates, the values were corrected for the contribution of K_2_HPO_4_ present in the suspension.

### 2.10. Statistical Analysis

All experiments were performed using 3–4 independent biological replicates, each analyzed in at least triplicate technical measurements. For each biological replicate, the mean value of the technical measurements was calculated. Data are presented as mean ± standard deviation (SD) of the biological replicates. Statistical differences between samples were evaluated using one-way analysis of variance (ANOVA) followed by Tukey’s post hoc test when applicable. Differences were considered statistically significant at *p* < 0.05.

Data obtained from mammalian cell experiments were analyzed using OriginPro 9.0 and are presented as mean ± standard error (SE). Statistical significance was evaluated using one-way analysis of variance (ANOVA) followed by Bonferroni’s multiple comparisons test, with *p* < 0.05 considered significant.

## 3. Results and Discussion

### 3.1. Sequential Recovery of Bioactive Compounds from SBY

Irreversible plasma membrane permeabilization is a prerequisite both for the efficient release of water-soluble intracellular compounds, including free amino acids, peptides, B-group vitamins, and antioxidants such as glutathione, and for enabling Alcalase to access the cell interior, thereby promoting protein hydrolysis.

In the present study, electropermeabilization was induced using a previously optimized protocol for SBY [39]. PEF treatment was performed at a flow rate of 140 mL/min. During their passage through the chamber, the cells were exposed to 10 monopolar rectangular electric pulses of 0.5 ms duration at an electric field strength of 3.1–3.25 kV/cm. The outlet temperature of the treated cell suspension varied between 35 and 38 °C and the specific treatment energy was 60.47 ± 2.10 kJ/L. Under these conditions, more than 99% of the cells were irreversibly permeabilized as detected by PI staining.

Following PEF treatment, the cell suspensions were incubated at room temperature for 4 h to allow the release of water-soluble intracellular compounds prior to the enzymatic hydrolysis step. Under these incubation conditions, most cellular proteins were retained in the cells [39]. Subsequently, the suspensions were centrifuged, and the residual biomass was subjected to enzymatic hydrolysis using Alcalase. This enzyme was selected based on our previous studies, which demonstrated its efficiency in hydrolyzing yeast proteins following electropermeabilization and its ability to generate peptide-rich fractions with high antioxidant activity [39,42].

For enzymatic hydrolysis, the resulting cell pellets were resuspended in 50 mM K_2_HPO_4_ to the original suspension volume, corresponding to 60.50 ± 1.63 mg DCW/mL, and Alcalase was added to a final concentration of 0.2% (*v*/*v*). Compared with our previous study [39], the biomass was approximately doubled while maintaining the same enzyme concentration. The cell suspensions were then incubated for 6 h at 40 °C with shaking at 100 rpm. The hydrolysis conditions followed the previously established protocol [39] enabling direct evaluation of the proposed cascade recovery strategy.

Heat treatment is a necessary step following enzymatic hydrolysis to ensure complete inactivation of the applied protease. Since water extracts, enzymatic hydrolysates, and cell lysates obtained by bead milling were compared in this study, both heated and unheated samples were analyzed to evaluate the effect of heat treatment and to enable direct comparison among the different sample types.

#### 3.1.1. Recovery of Bioactive Compounds from Brewer’s Yeast After Four Repitching Cycles

**Figure 1:**
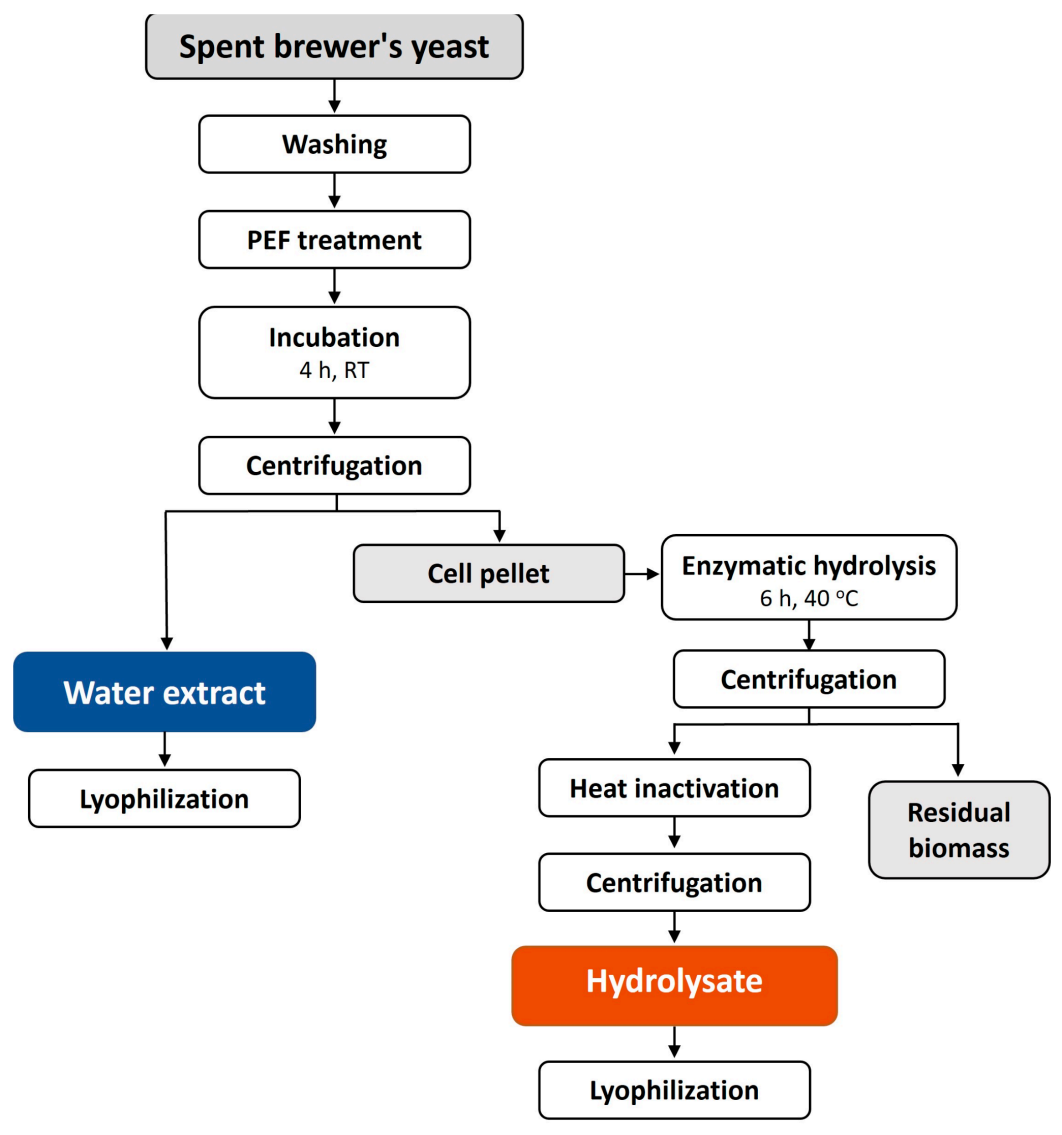
Schematic overview of the proposed PEF-assisted cascade extraction process. RT, room temperature.

The initial experiments were performed using yeast biomass obtained after four repitching cycles. Since electropermeabilization results from the generation of an additional transmembrane potential, it can occur only in cells with intact plasma membranes. In this study, the percentage of dead cells prior to PEF treatment was 11.6 ± 1.4%, confirming that most of the population could be effectively electropermeabilized. After PEF treatment and a 4 h incubation at room temperature, the released intracellular compounds were separated from the residual biomass by centrifugation, and the resulting cell pellets were frozen until enzymatic hydrolysis. A schematic overview of the cascade extraction procedure is presented in Figure 1.

**Figure 2:**
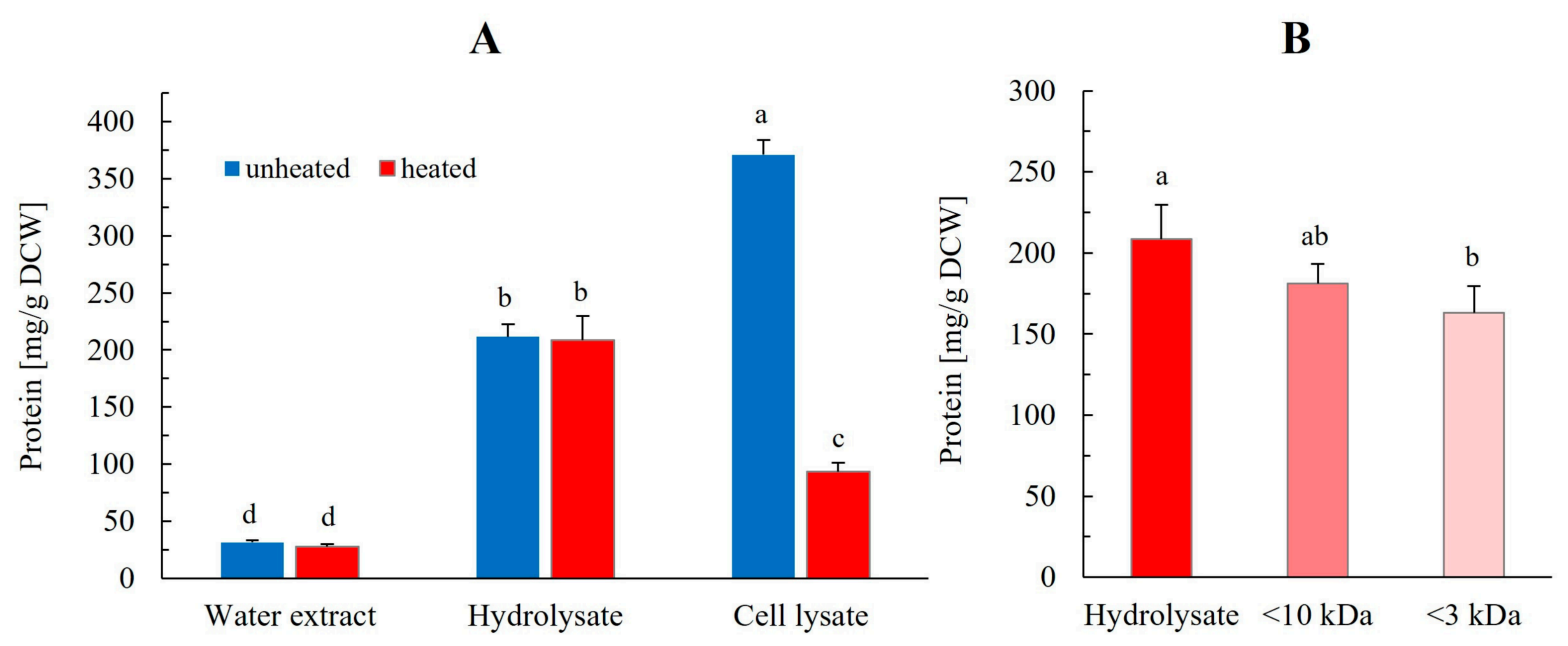
Protein content of the different fractions obtained from PEF-treated SBY and the cell lysate prepared from control cells by bead milling. (**A**) Protein content of water extract, hydrolysate, and cell lysate before and after heating at 90 °C for 15 min. (**B**) Protein content of heated hydrolysates before and after ultrafiltration using 10 and 3 kDa molecular weight cut-off membranes. Data are presented as mean ± SD (*n* = 4). Different lowercase letters indicate statistically significant differences according to one-way ANOVA followed by Tukey’s post hoc test (*p* < 0.05).

The distribution of protein among the different fractions obtained during the sequential extraction process is shown in Figure 2A. The unheated water extract contained a relatively small amount of protein (31.80 ± 3.16 mg/g DCW). Similar observations have previously been reported for both baker’s yeast and SBY, where incubation of electropermeabilized cells in water resulted in efficient release of low-molecular-weight intracellular compounds, while most proteins remained associated with the residual biomass [33,39]. This limited protein recovery may be attributed to protein aggregation resulting from the decrease in cytosolic ionic strength and pH following plasma membrane permeabilization, as well as to the barrier properties of the yeast cell wall, which restrict the release of macromolecules into the extracellular medium. The subsequent enzymatic hydrolysis yielded 211.45 ± 11.13 mg/g DCW, compared with 371 ± 13 mg/g DCW recovered after bead milling.

The total amount of protein recovered through the two sequential extraction steps reached approximately 243 mg/g DCW. This value is close to the protein yield previously obtained when Alcalase hydrolysis was applied directly to PEF-treated cells without prior removal of the released intracellular components (254 ± 17 mg/g DCW) [39]. It should be noted that no additional washing step was applied after hydrolysis to recover peptides retained within the residual biomass. Owing to the high biomass loading, a fraction of the generated peptides may have remained within the permeabilized cells and could be recovered by additional washing of the residual biomass. Heat treatment did not significantly affect the protein content of either the water extract or the hydrolysate, indicating a high degree of thermal stability. In contrast, a significant decrease in soluble protein was observed in the cell lysate, most likely as a result of heat-induced denaturation and precipitation of native proteins. The absence of a similar effect in the water extract and hydrolysate suggests that these fractions were enriched in low-molecular-weight, heat-stable peptides that remained soluble after heating. To further characterize the molecular weight distribution of the peptides present in the hydrolysate, the heated sample was subjected to sequential ultrafiltration through 10 and 3 kDa molecular weight cut-off membranes. As shown in Figure 2B, approximately 87% of the protein present in the heated hydrolysate consisted of peptides with molecular masses below 10 kDa, whereas peptides smaller than 3 kDa accounted for about 78% of the total protein. Thus, the protein fraction recovered after Alcalase treatment consists predominantly of low-molecular-weight peptides. These results are consistent with previous SDS-PAGE analyses of Alcalase-generated hydrolysates from baker’s and brewer’s yeast [39,42].

**Figure 3:**
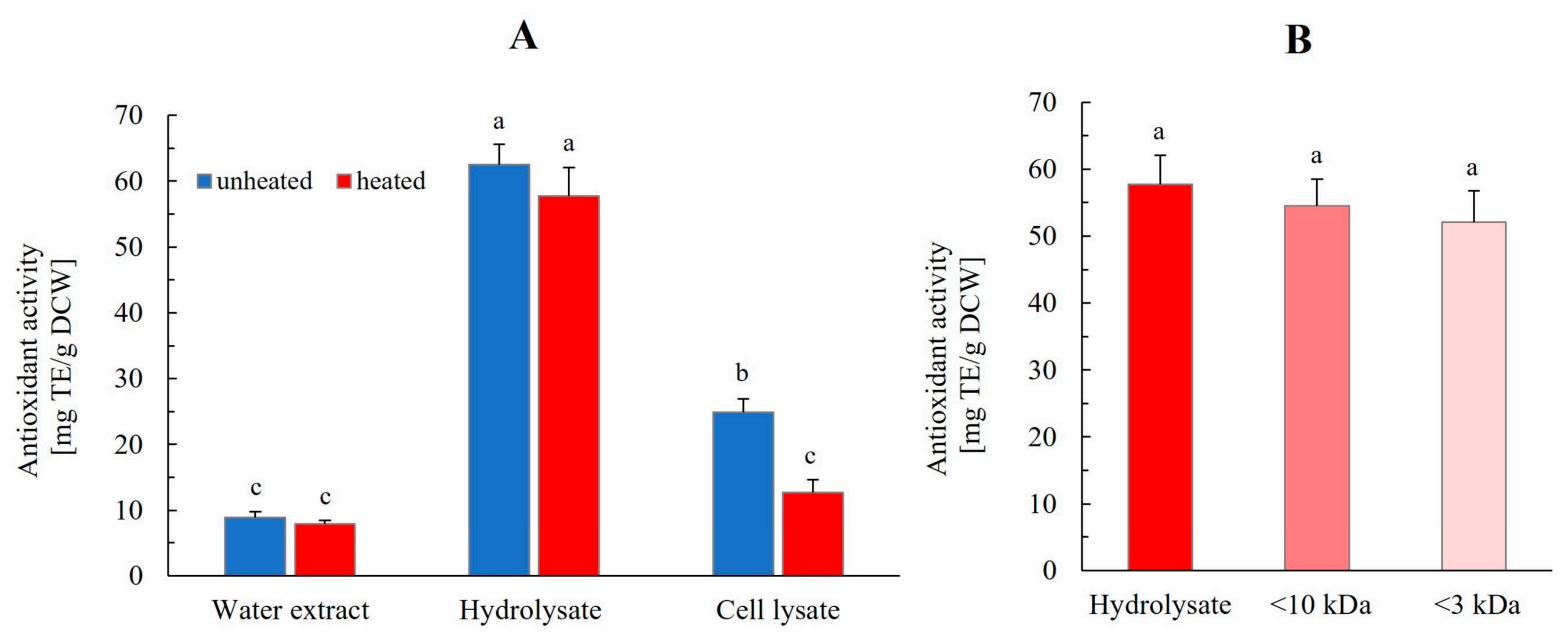
Antioxidant activity of the different fractions obtained from PEF-treated SBY and the cell lysate prepared from untreated control cells by bead milling. (**A**) Antioxidant activity before and after heating at 90 °C for 15 min. (**B**) Antioxidant activity of heated hydrolysates before and after ultrafiltration through 10 and 3 kDa molecular weight cut-off membranes. Data are presented as mean ± SD (*n* = 3). Different lowercase letters indicate statistically significant differences according to one-way ANOVA followed by Tukey’s post hoc test (*p* < 0.05).

A similar trend was observed for the antioxidant activity. The activity of the unheated water extract was 8.93 ± 0.85 mg TE/g DCW, whereas that of the hydrolysate before thermal inactivation was 62.52 ± 3.10 mg TE/g DCW (Figure 3A). As a result, the total activity recovered through the two sequential extraction steps reached approximately 71.45 mg TE/g DCW. This value was higher than that previously obtained by direct hydrolysis of PEF-treated cells (62.7 ± 9.3 mg TE/g DCW), suggesting that the cascade extraction strategy may improve the overall recovery of antioxidant compounds.

Thermal treatment resulted in slight reductions in the antioxidant activity of the water extract and the hydrolysate, although these differences were not statistically significant. In contrast, the activity of the cell lysate decreased by approximately 50%, in agreement with previous observations [39]. This pronounced reduction is consistent with the contribution of thermolabile intracellular antioxidant enzymes to the overall activity of the lysate, as indicated by the comparable values obtained for the water extract and the heated cell lysate. This suggests that the remaining activity may be due to the presence of heat-stable low-molecular-weight intracellular antioxidants.

Sequential ultrafiltration of the heated hydrolysate through 10 and 3 kDa membranes did not result in a statistically significant loss of antioxidant activity. The <3 kDa permeate retained 52.12 ± 4.65 mg TE/g DCW, corresponding to approximately 90% of the initial value (Figure 3B). These findings indicate that the antioxidant properties of the hydrolysate are largely associated with compounds smaller than 3 kDa.

One of the major limitations of the enzymatic hydrolysis of yeast biomass is the thick, poorly permeable cell wall, particularly in SBY obtained after multiple repitching cycles, which restricts the accessibility of intracellular proteins to exogenous proteases. Under suitable electrical conditions, PEF treatment induces structural changes in the outer mannoprotein layer of the cell wall, increasing its permeability to macromolecules even in stationary-phase cells [33,34,39]. This dual effect of PEF—alteration of plasma membrane integrity (electropermeabilization) together with increased cell wall permeability—results in the high efficiency of enzymatic hydrolysis observed both in the present study and in our previous studies.

**Figure 4:**
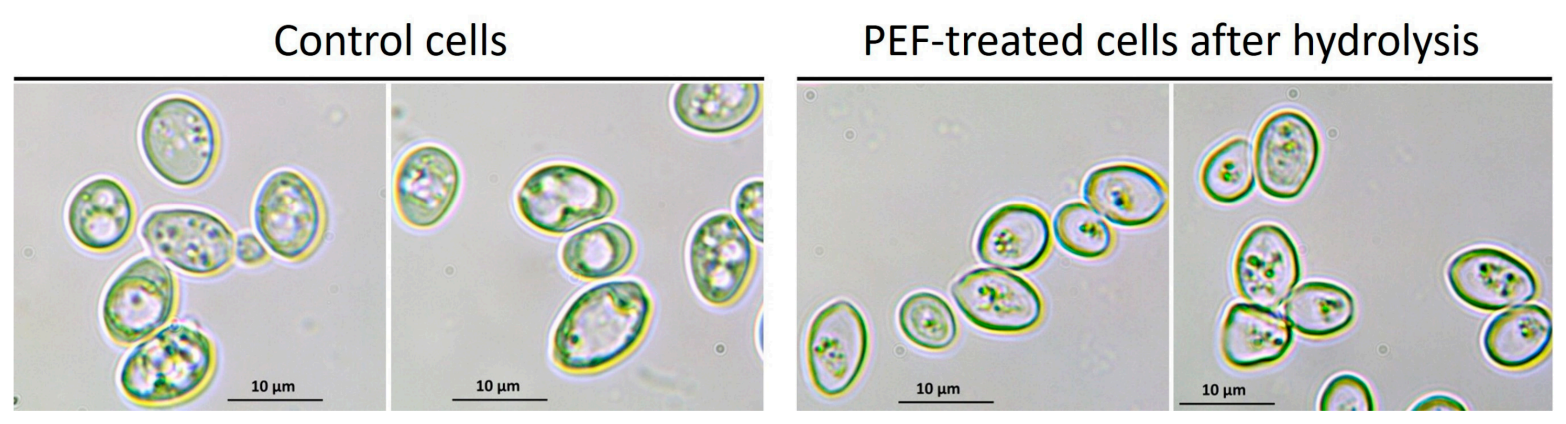
Light microscopy images of control yeast cells (**left**) and residual PEF-treated yeast cells following enzymatic hydrolysis (**right**), acquired using a 100×/1.25 oil-immersion objective.

Light microscopy images of untreated control yeast cells and residual PEF-treated cells after enzymatic hydrolysis are shown in Figure 4. Although the PEF-treated cells exhibited clear morphological alterations compared with the control cells, they remained structurally intact, with no evidence of cell fragmentation. This observation indicates that the β-glucan network responsible for the mechanical stability of the cell wall remained largely intact. Thus, in the proposed cascade valorization process, the yeast cell wall, which represents a major barrier to enzymatic hydrolysis under conventional conditions, becomes a technological advantage by serving as a natural filtration barrier. This allows the sequential recovery of two product fractions with distinct compositions: a water extract enriched in low-molecular-weight intracellular compounds, including free amino acids, small peptides, B-group vitamins, and antioxidants such as glutathione [39], and a hydrolysate with high antioxidant activity consisting predominantly of peptides below 3 kDa. The absence of cell fragmentation also facilitates the separation of the residual biomass from the hydrolysate by centrifugation, thereby simplifying downstream processing and providing an additional technological advantage of the proposed cascade extraction strategy.

#### 3.1.2. Recovery of Bioactive Compounds from Brewer’s Yeast Obtained After Six Repitching Cycles

To evaluate the applicability of the proposed PEF-assisted cascade extraction strategy to industrially relevant yeast biomass, additional experiments were performed using brewer’s yeast collected after six repitching cycles. At this stage, the biomass approaches the end of its technological lifetime and is therefore commonly removed from the brewing process. The proportion of non-viable cells prior to treatment, determined by propidium iodide staining, was 14.00 ± 1.73%, indicating that most cells remained viable despite the increased number of repitching cycles. The same cascade extraction protocol was applied as described above, except that the cell pellets obtained after PEF treatment, a 4 h incubation, and removal of the water-soluble fraction were subjected to enzymatic hydrolysis without an intermediate freezing step. In parallel, untreated control cells were subjected to the same incubation and hydrolysis procedure, allowing the contribution of PEF treatment to the recovery of proteins, free amino nitrogen (FAN), and antioxidant activity to be assessed.

**Figure 5:**
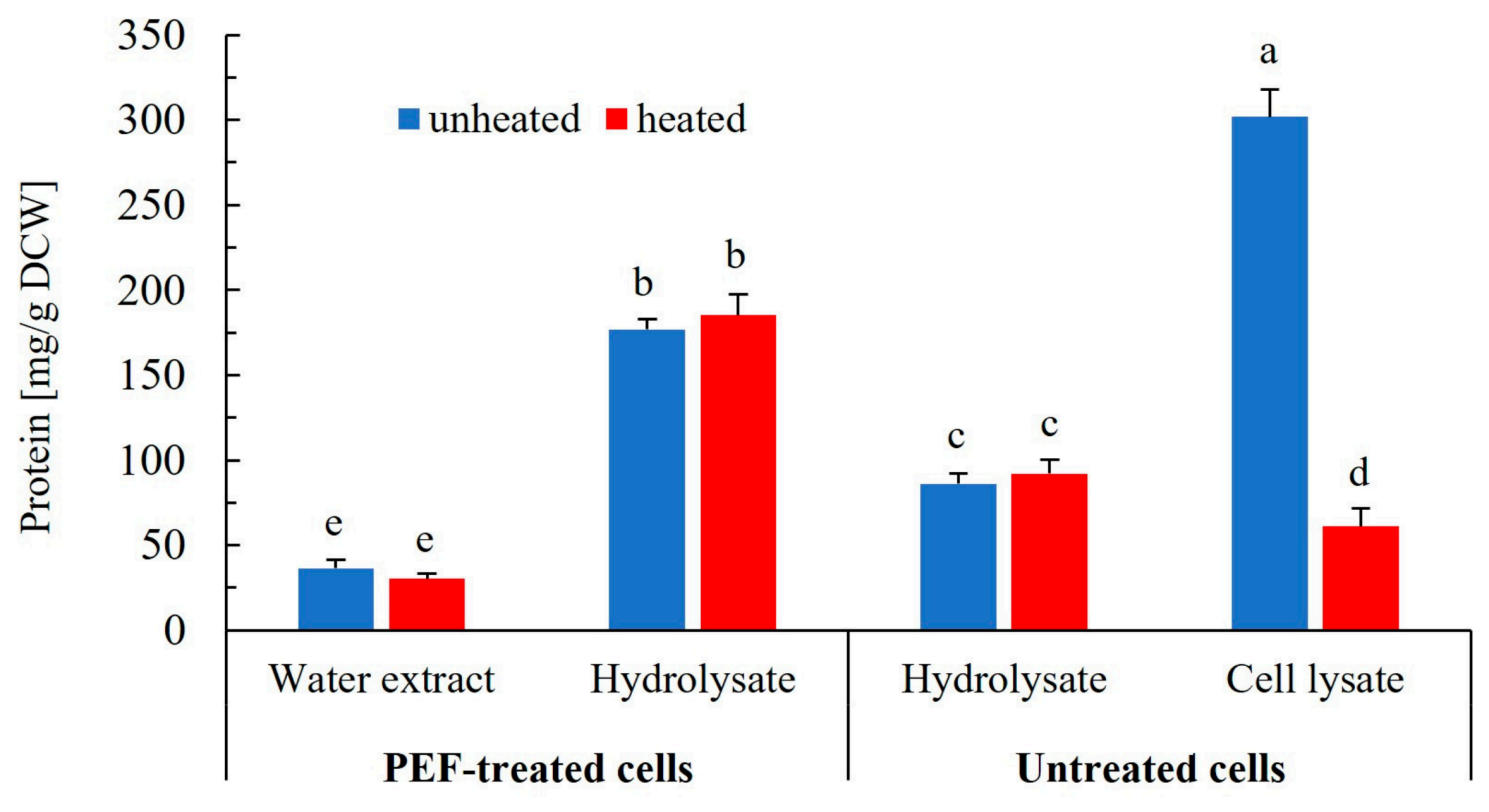
Protein content of the water extract, hydrolysates from PEF-treated and control cells, and the cell lysate prepared from control cells, before and after heating at 90 °C for 15 min. Data are presented as mean ± SD (*n* = 4). Different lowercase letters indicate statistically significant differences according to one-way ANOVA followed by Tukey’s post hoc test (*p* < 0.05).

The protein content of the non-heated cell lysate was 302.00 ± 14.53 mg/g DCW, which was lower than that measured in yeast collected after four repitching cycles; nevertheless, the overall pattern of protein recovery from PEF-treated cells (Figure 5) was similar to that observed for the corresponding biomass in the previous experiment (Figure 2). The protein contents of the water extract and the hydrolysate obtained from PEF-treated cells prior to heating were 36.72 ± 4.78 and 176.70 ± 6.02 mg/g DCW, respectively.

Thus, the overall protein recovery achieved by the sequential extraction procedure corresponded to approximately 70% of that obtained by mechanical cell disruption. The protein content of the hydrolysate obtained from PEF-treated cells was significantly higher (approximately two-fold) than that of the hydrolysate from control cells. Thermal treatment had no significant effect on the protein content of either the water extract or the hydrolysate derived from PEF-treated and control cells. On the other hand, as observed for yeast after four repitching cycles, it significantly reduced the protein content of the cell lysate.

**Figure 6:**
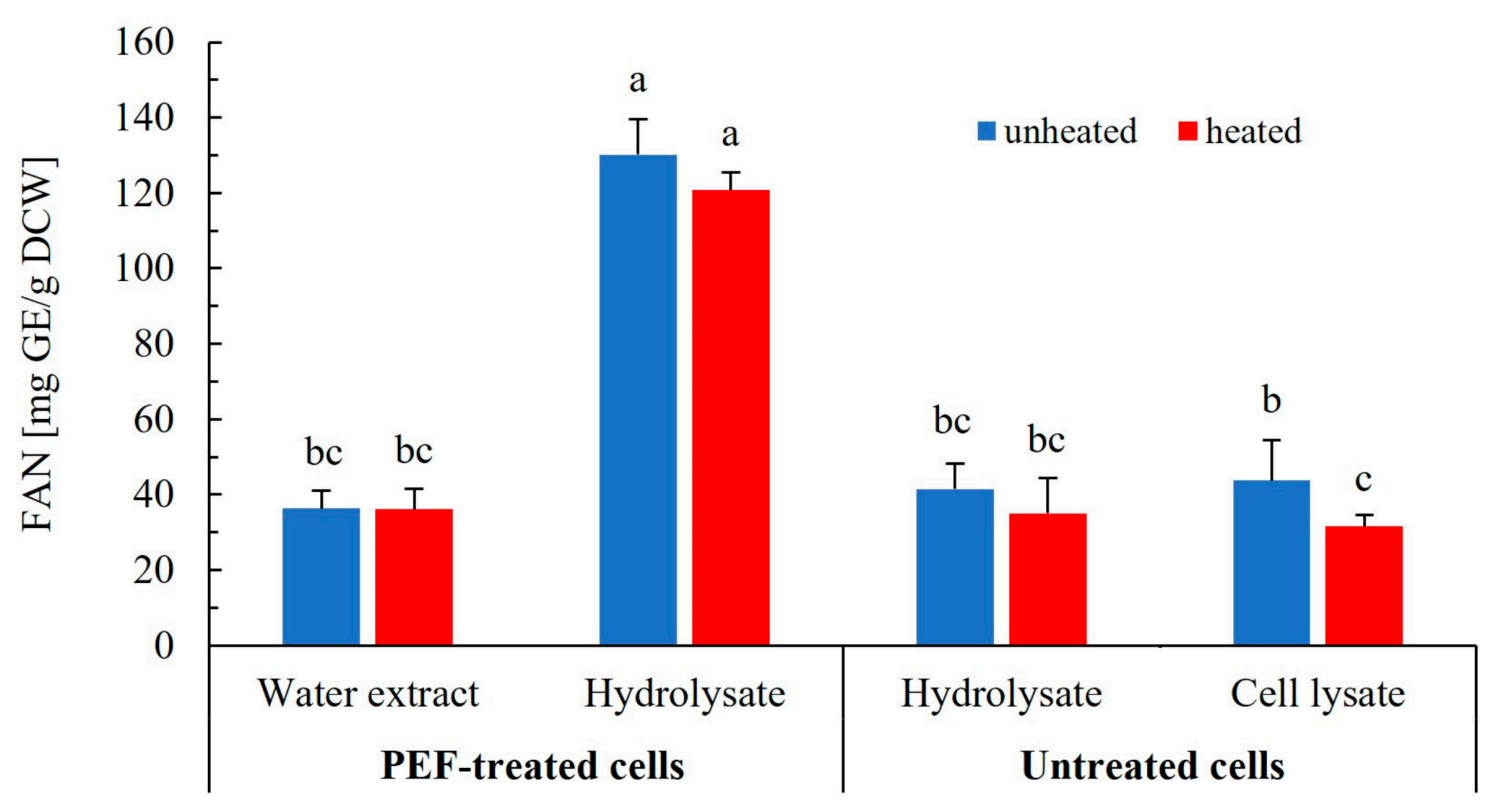
FAN content of the water extract, hydrolysates from PEF-treated and control cells, and the cell lysate prepared from control cells, before and after heating at 90 °C for 15 min. Data are presented as mean ± SD (*n* = 3). Different lowercase letters indicate statistically significant differences according to one-way ANOVA followed by Tukey’s post hoc test (*p* < 0.05).

The FAN content in the hydrolysate obtained from electropermeabilized yeast before heat treatment was 138.31 ± 9.66 mg GE/g DCW (Figure 6). When the amount recovered in the water extract (37.39 ± 4.02 mg GE/g DCW) was also taken into account, the overall yield was approximately 3.6-fold higher than that measured in the cell lysate (49.05 ± 9.90 mg GE/g DCW).

In addition, the hydrolysate obtained from PEF-treated cells had significantly higher FAN levels (3.17- and 3.44-fold, respectively) than that from control cells before and after heat treatment. Since FAN reflects the concentration of free α-amino groups, these results suggest a more extensive generation and accumulation of low-molecular-weight nitrogen-containing compounds, including free amino acids and short peptides, during enzymatic hydrolysis in PEF-treated cells.

**Figure 7:**
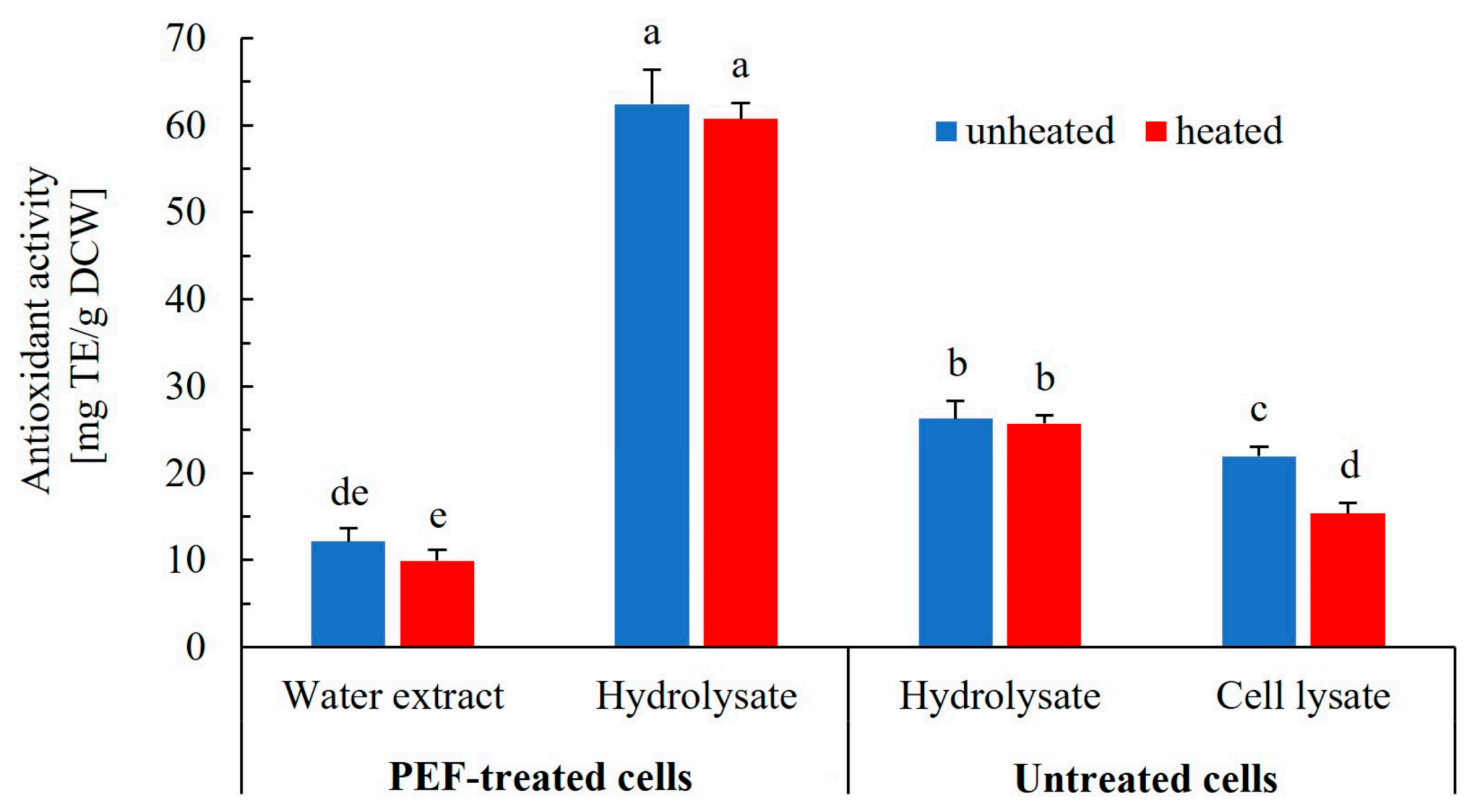
Antioxidant activity of the water extract and hydrolysates obtained from PEF-treated and control cells, and the cell lysate prepared from control cells, before and after heating at 90 °C for 15 min. Data are presented as mean ± SD (*n* = 3). Different lowercase letters indicate statistically significant differences according to one-way ANOVA followed by Tukey’s post hoc test (*p* < 0.05).

The antioxidant activity of the water extract obtained from PEF-treated cells before heat treatment was 11.35 ± 1.74 mg TE/g DCW, whereas that of the hydrolysate reached 62.40 ± 4.25 mg TE/g DCW (Figure 7). Consequently, the total activity recovered through the sequential extraction process reached approximately 74 mg TE/g DCW, exceeding the amount previously obtained by direct hydrolysis of PEF-treated cells [39]. The hydrolysate obtained from PEF-treated cells exhibited significantly higher activity than that obtained from control cells, whereas thermal treatment had no significant effect on either the water extract or the hydrolysates. In contrast, it significantly reduced the activity of the cell lysate, as also observed for the yeast collected after four repitching cycles (Figure 3).

The enhanced recovery achieved by the proposed PEF-assisted cascade extraction strategy may be attributed to the formation of two distinct antioxidant fractions. During the initial extraction step, water-soluble intracellular antioxidants are recovered as a separate fraction. Glutathione, which is efficiently released from electropermeabilized yeast cells, may represent one of such examples [33,39,48]. Subsequent Alcalase hydrolysis of the residual biomass provides an additional source of antioxidant compounds. As a result, the combined activity of the water extract and the hydrolysate exceeded that obtained by direct hydrolysis alone. These findings indicate that the proposed PEF-assisted cascade extraction strategy enables more efficient utilization of the antioxidant potential of brewer’s yeast through the sequential recovery of low-molecular-weight intracellular antioxidants and peptide-rich hydrolysates.

The dry matter recovered in the non-heated water extract was 146.58 ± 4.26 mg/g DCW. Since no enzyme was added during this initial step, enzyme inactivation was not required, allowing the dry matter to be determined in its native, non-heated form. The dry matter recovered in the hydrolysate fraction obtained from PEF-treated cells was 385.89 ± 11.67 mg/g DCW. It was determined after thermal inactivation of Alcalase and subsequent centrifugation, as both steps are required to obtain the final hydrolysate. The protein, FAN, and antioxidant activity expressed per gram of recovered dry matter are summarized in Table 1. The hydrolysate was characterized by high protein content (480.18 ± 34.75 mg/g dry matter).

The ABTS radical scavenging activity reached 157.37 ± 7.02 mg TE/g dry matter, comparable to that reported for peptide-rich yeast extracts obtained by membrane-based fractionation and purification processes [49]. This high activity may be attributed, at least in part, to the predominance of low-molecular-weight peptides. Ultrafiltration experiments showed that approximately 90% of the antioxidant activity was retained in the <3 kDa fraction, which also contained most of the soluble protein. Compared with the water-soluble fraction, the hydrolysate exhibited substantially higher protein content and antioxidant activity, whereas the increase in FAN was more moderate, consistent with the endoprotease activity of Alcalase. Low-molecular-weight peptides are of particular interest because numerous studies have reported enhanced biological activities, including antioxidant and ACE-inhibitory properties, compared with higher-molecular-weight fractions [18]. These findings further highlight the potential of the proposed cascade process for the production of a peptide-rich fraction with high antioxidant potential.

### 3.2. Effect of the Recovered Water Extract on Mammalian Cell Viability

Although the water extract contained substantially lower amounts of dry matter than the corresponding hydrolysate, previous studies have demonstrated that it is enriched in intracellular water-soluble compounds, including B-group vitamins, free amino acids, peptides, and antioxidants such as glutathione, while containing relatively low levels of purines compared with the mechanically obtained cell lysate [33,39]. These properties make the recovered fraction an attractive alternative to whole brewer’s yeast biomass as a source of vitamins, antioxidants, and other bioactive compounds, suggesting its potential applicability as a functional food ingredient or nutraceutical supplement.

**Figure 8:**
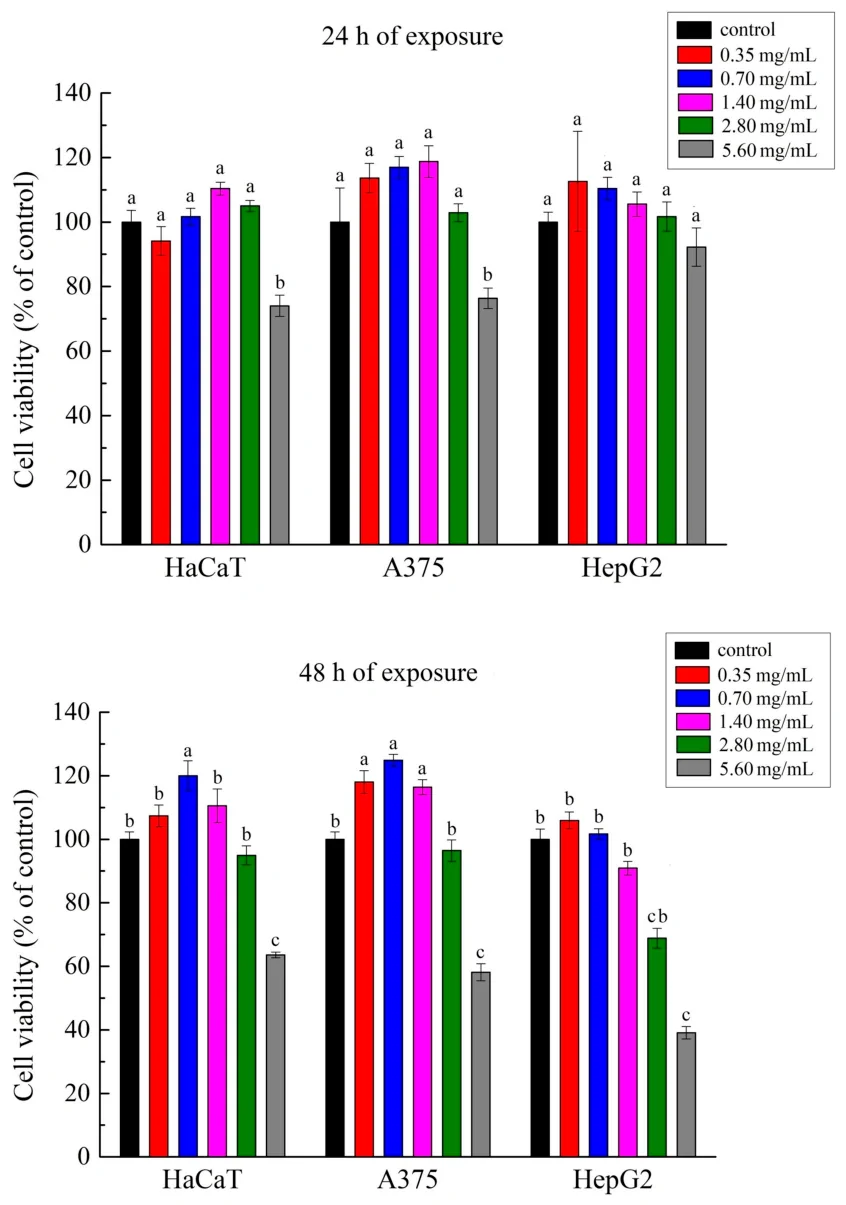
Effect of the water extract obtained after PEF treatment on the viability of HaCaT, A375, and HepG2 cells following 24 and 48 h of exposure. Cell viability is expressed as a percentage of the untreated control. Data are presented as mean ± SE (*n* = 3). Different lowercase letters indicate statistically significant differences between groups (one-way ANOVA followed by Bonferroni’s multiple comparisons test, *p* < 0.05).

To obtain a preliminary assessment of its biological compatibility, the effects of the recovered fraction on mammalian cell viability were evaluated using three different cell lines: HepG2, HaCaT, and A375. Cell viability was determined after 24 and 48 h of exposure to lyophilized water extracts reconstituted in DMEM using the crystal violet assay. As shown in Figure 8, after 24 h, a tendency toward increased cell viability was observed at low and intermediate extract concentrations (0.35–2.80 mg dry matter/mL) in all three cell lines, although none of these effects reached statistical significance.

At the highest concentration tested, cell viability was significantly reduced in HaCaT and A375 cells. After 48 h, a biphasic response was observed. Cell viability increased significantly in HaCaT cells at 0.70 mg dry matter/mL and in A375 cells at 0.35–1.40 mg dry matter/mL, whereas HepG2 cells showed no significant stimulatory response. At higher concentrations, the stimulatory effect gradually diminished. A significant reduction below the control level was observed at ≥2.80 mg dry matter/mL in HepG2 cells and only at 5.60 mg dry matter/mL in HaCaT and A375 cells. Overall, the water extract was well tolerated within a defined concentration range and the stimulatory effect observed at lower concentrations most probably resulted from the enrichment of the culture medium with free amino acids, B-group vitamins, glutathione and other low-molecular-weight intracellular constituents present in the water extract. The greater sensitivity of HepG2 cells may be related to their liver-derived phenotype and higher metabolic activity, making them more responsive to increased concentrations of soluble extract constituents. The reduced viability observed at the highest concentrations may reflect the cellular response to hypertonic conditions caused by the elevated ionic content of the reconstituted extracts rather than the intrinsic toxicity of the recovered compounds. These findings support further biological evaluation of the recovered fraction for food and nutraceutical applications.

The results of this study demonstrate the feasibility of a cascade extraction strategy for the valorization of SBY biomass. The proposed approach combines recovery of water-soluble intracellular compounds released following irreversible electropermeabilization with subsequent enzymatic hydrolysis of the residual biomass, enabling efficient production of protein-rich hydrolysates. An advantage of the proposed protocol is that both PEF treatment and post-pulse incubation are performed in water. The low conductivity of the medium minimizes Joule heating, allowing efficient processing of highly concentrated cell suspensions without substantial temperature increase during pulse application. Under the selected electrical conditions, the fraction of irreversibly permeabilized cells exceeded 99%, while the outlet temperature remained between 35 and 38 °C. Consequently, the electrical treatment can be carried out at room temperature without cooling or temperature adjustment of the cell suspension, simplifying the process. A substantial proportion of low-molecular-weight intracellular compounds, including FAN and antioxidants, was recovered by incubating the treated cells in water without buffers or added salts. Removal of the water-soluble fraction prior to hydrolysis enabled the production of two distinct fractions while maintaining overall protein and FAN yields comparable to those previously obtained by direct hydrolysis of PEF-treated cells. A further advantage of this approach is that thermolabile intracellular bioactive compounds, such as glutathione and B-group vitamins previously identified in this fraction [39], can be recovered before enzymatic hydrolysis, thereby avoiding exposure to the hydrolysis conditions and the subsequent enzyme inactivation step (90 °C, 15 min). Notably, the combined antioxidant activity of the water extract and hydrolysate fractions showed a trend toward higher values than that previously obtained by direct hydrolysis alone.

After recovery of the water-soluble intracellular compounds, the remaining biomass was readily hydrolyzed with Alcalase, demonstrating that the preliminary extraction step did not compromise the efficiency of the subsequent enzymatic hydrolysis. PEF treatment eliminates the need for both mechanical cell disruption and the use of cell wall-degrading enzymes prior to hydrolysis. Irreversible membrane electropermeabilization together with the PEF-induced increase in cell wall porosity facilitates Alcalase access to intracellular proteins. These structural changes are particularly important for SBY, whose cell walls become progressively less permeable during successive repitching cycles, resulting in substantially longer hydrolysis times than those required for non-repitched yeast [14].

Hydrolysis of PEF-treated biomass was highly efficient even with a relatively short incubation time and at a temperature well below the optimum for Alcalase. Compared with control cells hydrolyzed under identical conditions, PEF pretreatment resulted in approximately two-fold higher protein recovery, 3.44-fold higher FAN release, and 2.36-fold higher antioxidant activity. These results are comparable to those reported in studies employing considerably more intensive hydrolysis conditions, including higher enzyme concentrations or combinations of different proteases, longer incubation times (12–48 h), and elevated temperatures (50–55 °C) [12,13,14].

The composition and potential applications of yeast-derived products are strongly influenced by both the characteristics of the starting biomass and the processing method employed [5]. Autolysis remains one of the most widely used and cost-effective methods for producing commercial yeast extracts for various applications [50,51,52]. However, because the process relies exclusively on endogenous hydrolytic enzymes, it generally requires 24–72 h of incubation at 45–60 °C and provides limited control over the extent of protein hydrolysis and the molecular weight distribution of the resulting peptides [14]. Additional drawbacks include difficulties in separating the soluble fraction from the residual solids and an increased risk of microbial contamination associated with the extended processing time. Prolonged incubation may also promote the degradation of labile bioactive compounds, including antioxidants and vitamins [8,53]. Various approaches have therefore been explored to accelerate autolysis, including chemical treatment [54], mechanical disruption [55,56], and physical pretreatments such as PEF [48,57,58]. Although PEF promotes autolysis through vacuolar membrane destabilization and the release of endogenous hydrolytic enzymes, recovering approximately 70% of the protein yield obtained after mechanical cell disruption still requires 24–48 h of incubation.

When the objective is the production of peptide-rich hydrolysates with diverse bioactivities, enzymatic hydrolysis using selected proteases is a well-established approach. The ability to control the extent of hydrolysis and the characteristics of the resulting fractions make it particularly suitable for the production of bioactive peptides [6,7,14]. Alcalase is a widely used commercial protease owing to its high catalytic efficiency, broad substrate specificity, and preference for cleaving peptide bonds adjacent to hydrophobic residues [59]. These properties make this enzyme particularly suitable for the hydrolysis of SBY proteins, which contain a substantial proportion of hydrophobic and aromatic amino acid residues frequently associated with peptide bioactivity [60]. Furthermore, in PEF-treated yeast biomass, the relatively low molecular weight of Alcalase provides an additional advantage by facilitating access to intracellular protein substrates, as previously demonstrated [39,42].

Although the present study demonstrates the feasibility of the proposed cascade extraction strategy, further research is needed to better characterize the recovered fractions. Antioxidant activity was evaluated using the ABTS assay, which is one of the most commonly used methods for determining antioxidant capacity. Nevertheless, complementary approaches would provide a more detailed characterization of their antioxidant properties. In addition, investigations aimed at identifying other biological activities would provide a more complete basis for assessing the potential applications of these fractions in areas such as functional foods, cosmetics, and pharmaceuticals.

The data presented here suggest that PEF pretreatment represents a promising approach for improving the enzymatic hydrolysis of SBY, thereby facilitating its valorization. Further development of the proposed cascade extraction strategy may focus on increasing the biomass-to-enzyme ratio and optimizing the enzymatic hydrolysis conditions, including incubation temperature and duration, to improve process efficiency while maintaining high protein recovery and antioxidant activity.

## 4. Conclusions

The findings of this study demonstrate that PEF-induced irreversible electropermeabilization combined with enzymatic hydrolysis represents a promising strategy for the cascade valorization of SBY biomass, enabling the sequential recovery of water-soluble intracellular compounds and antioxidant-rich protein hydrolysates. PEF pretreatment markedly enhanced the efficiency of enzymatic hydrolysis under relatively mild operating conditions, yielding results comparable to those achieved using substantially more intensive hydrolysis processes. The use of continuous-flow PEF technology together with a commercially available protease provides a technically simple, scalable process with good potential for industrial implementation.

## Figures and Tables

**Table 1:** Protein content, FAN, and antioxidant activity of the water extract and the hydrolysate from PEF-treated cells, on a dry matter basis.

Fraction	Protein mg/g Dry Matter	FAN mg GE/g Dry Matter	Antioxidant Activity mg TE/g Dry Matter
Water extract	250.50 ± 33.30	255.10 ± 28.31	77.43 ± 12.08
Hydrolysate	480.18 ± 34.75	316.53 ± 13.35	157.37 ± 7.02

Water extract was characterized without thermal treatment; dry matter, protein, FAN, and antioxidant activity in the hydrolysate were determined after thermal inactivation of Alcalase (90 °C for 15 min).

## Data Availability

The original contributions presented in this study are included in the article. Further inquiries can be directed to the corresponding author.
